# Agephagy – Adapting Autophagy for Health During Aging

**DOI:** 10.3389/fcell.2019.00308

**Published:** 2019-11-28

**Authors:** Eleanor R. Stead, Jorge I. Castillo-Quan, Victoria Eugenia Martinez Miguel, Celia Lujan, Robin Ketteler, Kerri J. Kinghorn, Ivana Bjedov

**Affiliations:** ^1^UCL Cancer Institute, University College London, London, United Kingdom; ^2^Section on Islet Cell and Regenerative Biology, Joslin Diabetes Center, Boston, MA, United States; ^3^Department of Genetics, Harvard Medical School, Boston, MA, United States; ^4^MRC Laboratory for Molecular Cell Biology, University College London, London, United Kingdom; ^5^Institute of Healthy Ageing, University College London, London, United Kingdom; ^6^Department of Genetics, Evolution and Environment, University College London, London, United Kingdom; ^7^Institute of Neurology, University College London, London, United Kingdom

**Keywords:** autophagy, aging, target of rapamycin, insulin/IGF-1 signaling, proteostasis, DNA damage, mitophagy, anti-aging drugs

## Abstract

Autophagy is a major cellular recycling process that delivers cellular material and entire organelles to lysosomes for degradation, in a selective or non-selective manner. This process is essential for the maintenance of cellular energy levels, components, and metabolites, as well as the elimination of cellular molecular damage, thereby playing an important role in numerous cellular activities. An important function of autophagy is to enable survival under starvation conditions and other stresses. The majority of factors implicated in aging are modifiable through the process of autophagy, including the accumulation of oxidative damage and loss of proteostasis, genomic instability and epigenetic alteration. These primary causes of damage could lead to mitochondrial dysfunction, deregulation of nutrient sensing pathways and cellular senescence, finally causing a variety of aging phenotypes. Remarkably, advances in the biology of aging have revealed that aging is a malleable process: a mild decrease in signaling through nutrient-sensing pathways can improve health and extend lifespan in all model organisms tested. Consequently, autophagy is implicated in both aging and age-related disease. Enhancement of the autophagy process is a common characteristic of all principal, evolutionary conserved anti-aging interventions, including dietary restriction, as well as inhibition of target of rapamycin (TOR) and insulin/IGF-1 signaling (IIS). As an emerging and critical process in aging, this review will highlight how autophagy can be modulated for health improvement.

## The New Biology and Hallmarks of Aging

Aging is characterized by progressive deterioration of tissues and organs, leading to loss of physiological function and increased risk of death. In developed societies, we are witnessing a constant increase in the size of elderly populations, and an ensuing increase in people suffering from age-related diseases, making health improvement during aging an important challenge and a priority (Rae et al., 2010). Over the last few decades, outstanding progress has been made toward understanding the aging process. Specifically, we now know that, despite all of the complexities of aging, a single mutation in just one of a few genes in nutrient-sensing pathways is sufficient to extend lifespan in all model organisms tested (Kenyon, 2010; Partridge, 2010). Moreover, the effect of anti-aging mutations is evolutionary conserved from yeast to mammals, and importantly, the long-lived mutants in all model organisms are healthier (Fontana et al., 2010; Selman and Withers, 2011; Singh et al., 2019). Thus, an improved understanding of the underlying mechanisms of aging based on genetic findings, with translation into pharmacological interventions, has the potential to improve health in the continuously growing elderly populations of modern societies. The hope is that such strategies will at the same time prevent age-related diseases (Niccoli and Partridge, 2012; Kennedy et al., 2014; Partridge et al., 2018). Examples of this approach include the anti-diabetic and the anti-aging drug metformin, which is the first drug to be tested for improvement of various health parameters in elderly people (Barzilai et al., 2016), and rapamycin, which has been shown to improve the efficacy of the flu vaccination in aged individuals (Mannick et al., 2018). Such approaches, if successful in slowing the aging process and age-related diseases, would be expected to have a significant impact on the quality of life of elderly individuals, as well as an important socio-economical benefit (Rae et al., 2010; Partridge et al., 2018; Campisi et al., 2019).

Currently there are nine proposed and well-defined primary hallmarks of aging (Lopez-Otin et al., 2013) that contribute to cellular injury and damage. These comprise genomic instability, telomere attrition, and epigenetic alteration, loss of proteostasis, deregulated nutrient-sensing, mitochondrial dysfunction and cellular senescence. In addition, two integrative hallmarks, stem cell exhaustion and altered intercellular communication, lead to functional deterioration and aging phenotypes.

In this review we focus on the autophagy process, whose upregulation is a common denominator of all major pro-longevity interventions (Hansen et al., 2018), including dietary restriction and mild down-regulation of the nutrient-sensing pathways – insulin (IIS) and mechanistic target-of-rapamycin signalling (mTOR) (Lopez-Otin et al., 2013). We first consider the regulators and effectors of autophagy, and examine the role of autophagy in the aging process. We next focus on how autophagy regulates those processes affected by aging, such as proteostasis, the maintenance of genomic integrity and organelle degradation. Finally, we explore the potential therapeutic role of autophagy modulation in preventing the aging process and age-related diseases.

## Autophagy Pathway

Under normal physiological conditions autophagic is indispensable for cellular homeostasis, and is upregulated under stress conditions. One of the most common examples of a stress condition is starvation, where active autophagy enables survival by degrading cellular components (Mizushima, 2018; Lahiri et al., 2019). There are three different types of autophagy: macroautophagy, microautophagy and chaperone-mediated autophagy (CMA), and they differ on how cargo is delivered to lysosomes for degradation. Macroautophagy is the principal and most commonly studied type of autophagy that is described below, and is commonly referred to as autophagy (Ktistakis and Tooze, 2016). Microautophagy involves the cytosolic sequesteration of cellular debris by a small invagination of the lysosomal membrane, thereby accessing lysosomal enzymes for degradation. Lastly, CMA relies on the cytosolic heat shock cognate 70 (hsc70) chaperone to recognize a KFERQ motif in target cytosolic proteins, facilitating lysosomal degradation (Kaushik and Cuervo, 2018; Scrivo et al., 2018).

**FIGURE 1:**
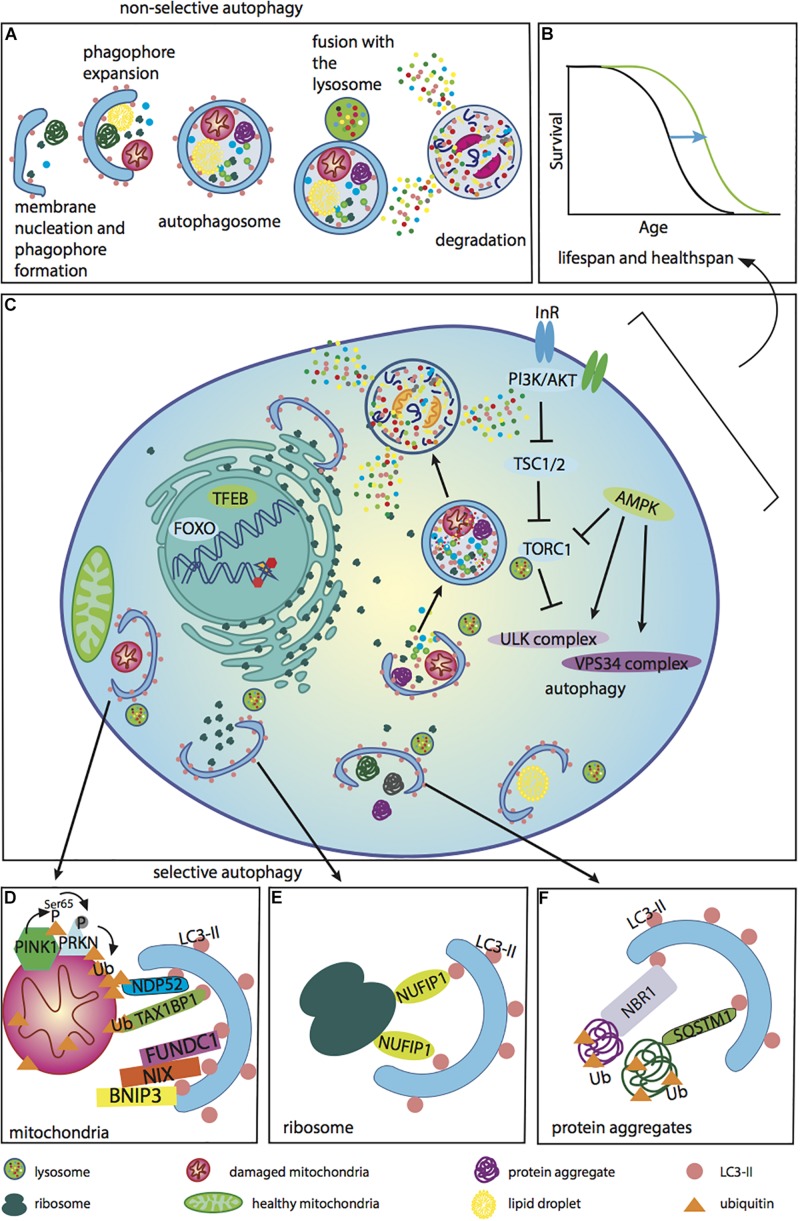
Autophagy regulators and functions associated with anti-aging effects. Different cellular components can be degraded by either non-selective or selective autophagy. These forms of autophagy are regulated by PI3K, mTOR, and AMPK, as well as the transcription factors TFEB and FOXO, which are accountable for the transcription of many genes involved in the autophagy process. **(A)** The different steps of non-selective autophagy. Upon autophagy initiation, a phagophore is formed and expanded, thereby producing an autophagosome. The matured phagosome fuses with the lysosome, initiating degradation of the autophagosome’s inner membrane. Cellular components captured within are subsequently degraded and released into the cytoplasm. Autophagy occurs under basal conditions in the cell, but can be up-regulated during stress. The autophagy process is inefficient during aging. **(B)** A lifespan curve illustrating the positive effects of enhancing autophagy during aging to improve the recycling of different cellular components, extending lifespan and healthspan. Anti-aging effects of autophagy up-regulation have been demonstrated in several model organisms. It has been shown in a number of studies that the lifespan of long-lived mutants is (reduced upon down-regulation of autophagy. In addition, there are a few critical studies showing that up-regulation of autophagy by overexpression of one of the autophagy genes extends lifespan. **(C)** An illustrative diagram demonstrating the negative regulation of autophagy by PI3K/AKT and mTORC1, and the positive modulation of autophagy by AMPK via effects on the autophagy complexes ULK and VPS34. Phagophore expansion leads to the engulfment of various cellular components, such as ribosomes, protein aggregates and lipids, via selective as well as non-selective autophagy. In addition, autophagy plays an important role in the DNA damage response and the nuclear-associated genes, TFEB and FOXO, play important roles in regulating autophagy. **(D)** Defective mitochondria are cleared by the cell through mitophagy. This is the process whereby damaged depolarized mitochondria can be degraded by the PINK1-Parkin pathway, which is ubiquitin-dependent. In cells with healthy mitochondria, PINK1 is continuously degraded, while Parkin is in the cytoplasm. Upon stress, PINK1 is stabilized on the outer mitochondrial membrane, where it phosphorylates ubiquitin and E3 ubiquitin ligase Parkin. Once Parkin is recruited to the mitochondria, it then ubiquitinates some of the outer membrane mitochondrial proteins. These polyubiquitin K63-linked chains are phosphorylated, creating a degradative signal for autophagy. Receptor proteins involved in this pathway include NDP52 and TAX1BP1. These proteins recognize phosphorylated polyubiquitin chains and link damaged mitochondria to LC3-II. Another mechanism for mitochondrial degradation involves receptor-mediated autophagy by BNIP3, NIX and FUNDC1. These receptors also interact with LC3 via the LIR domain and target depolarized mitochondria for degradation. For mitophagy to occur, damaged mitochondria must be separated by fission. Healthy mitochondria are essential for cellular ATP production and the maintenance of cellular energy homeostasis. **(E)** The ribophagy receptor NUFIP1 mediates degradation of ribosomes (Wyant et al., 2018). **(F)** Protein aggregates are ubiquitinated and degraded by autophagy with the help of the autophagy receptors NBR1 and SQSTM1.)

A critical feature of autophagy is the ability to degrade cellular components not only randomly, but also selectively, and the number of autophagy receptor proteins that deliver certain cargo for autophagic degradation is continually growing (Kirkin, 2019). For selective autophagy to occur, LIR (LC3-interacting region)-containing receptors are essential and link specific cargo with LC3-II proteins on autophagosomes. The number of identified selective autophagy cargos are expanding; examples include degradation of glycogen by glycophagy, ferritin by ferrinophagy, protein aggregates by aggrephagy, lipids by lipophagy, and ribosomes by ribophagy. Organelle degradation is referred to as pexophagy for peroxisomes, mitophagy for mitochondria, and reticulophagy for endoplasmic reticulum (Dikic, 2017). This selective degradation is critical for ridding the cell of damaged constituents (Figure 1).

The autophagy process depends on the formation of different complexes on the membrane and can be divided into a number of steps: initiation, nucleation, elongation, autophagosome-lysosome fusion and degradation of sequestered material (Ktistakis and Tooze, 2016; Galluzzi et al., 2017; Lahiri et al., 2019). The Atg1/ULK1 complex, which is regulated by mTOR and AMPK, initiates the formation of a complex that regulates phagophore membrane nucleation at the endoplasmic reticulum and possibly other membrane enclosed organelles (Carlsson and Simonsen, 2015). The ATG1/ULK1 complex further activates the phosphatidylinositol 3-kinase (PI3K) complex through a series of phosphorylation events, which incorporates phosphatidylinositol 3-phosphate into the phagophore to form an autophagosome (Ktistakis et al., 2014). Two conjugation reactions are critical for the elongation of the autophagosome and closure. First, the conjugation of ATG12 to ATG5, and its interacting partner ATG16L1, via the ubiquitin-conjugation like enzymes ATG7 and ATG10, generates an ATG5-12/16L1 complex that is required for mediating the linkage of ATG8/LC3/GABARAP protein family to the autophagosome membrane (Nakatogawa, 2013; Lahiri et al., 2019). Following cleavage of pro-ATG8/LC3/GABARAP by the ATG4 family of proteases, and a two-step conjugation reaction requiring ATG3 and ATG7, the cleaved form of ATG8/LC3/GABARAP is anchored to phosphatidylethanolamine (PE) in the inner and outer autophagosome membrane. ATG8-PE/LC3-GABARAP can also be removed from the autophagosome membrane by the ATG4 family of proteins, although the relevance of this removal in mammalian cells is not clear (Kauffman et al., 2018; Agrotis et al., 2019). Recent data seems to suggest that ATG8-PE/LC3/GABARAP-II is required for late stages of autophagy (fusion and degradation of inner autophagosomal membrane) (Nguyen et al., 2016; Tsuboyama et al., 2016). In addition, ATG8/LC3/GABARAP can bind to selective autophagy receptors, such as SQSTM1/p62, that bind to ubiquitinated proteins and organelles to mediate selective cargo degradation (Kirkin, 2019). Membrane specific LC3-II and p62 are the most commonly used autophagy markers (Klionsky et al., 2012). Once formed, autophagosomes fuse with lysosomes to form autophagolysosomes (Nakamura and Yoshimori, 2017). It is in this latter structure that unwanted cellular components are degraded by acidic lysosomal hydrolases. Autophagy is a highly coordinated and dynamic process that is tightly controlled by post-translational regulation in a spatio-temporal manner (Botti-Millet et al., 2016; Pengo et al., 2017; Delorme-Axford and Klionsky, 2018), and perturbation in any step can lead to the accumulation of undigested material and aggregates.

## Autophagy Regulators and Down-Stream Effectors and Their Links to Aging

Various cellular energy and metabolic sensors act as major regulators of autophagy, with their coordinated action leading to different autophagy activities, thereby maintaining cellular homeostasis (Table 1). Activated AMP kinase (AMPK) induces autophagy in response to low ATP/AMP cellular energy status. Several underlying mechanisms have been described for AMPK-mediated autophagy up-regulation, such as phosphorylation of ACSS2 (acetyl-CoA synthetase short-chain family member 2). AMPK can also alter autophagy via induction of TFEB (transcription factor EB)-mediated transcription of lysosomal and autophagy genes (Li X. et al., 2017) and via inhibition of the TOR pathway (Saxton and Sabatini, 2017). Furthermore, AMPK phosphorylates BECN1 and PIK3C3/VPC34, as well ULK1, to stimulate autophagic function (Hardie et al., 2016). Overexpression of AMPK in the *Drosophila* nervous system induces autophagy in the brain and cell non-autonomously in the intestine, resulting in lifespan extension (Ulgherait et al., 2014). AMPK is a complex sensor of cellular energy status and can be activated genetically, or pharmacologically by metformin, leading to lifespan extension in organisms ranging from yeast to mice (Pryor and Cabreiro, 2015; Salminen et al., 2016). Detailed worm studies uncovered metformin-induced alterations of microbial metabolism (Cabreiro et al., 2013), specifically the production of bacterial agmatine that regulates lipid metabolism and promotes lifespan extension (Pryor et al., 2019).

**TABLE 1:** Autophagy regulators and down-stream effectors.

**Autophagy regulator**	**Organism**	**Longevity**	**Blocked by autophagy impairment?**	**Down-stream effectors and mechanisms**	**References**
mTOR	*Caenorhabditis elegans*	+∼20% (mean lifespan) *daf-*15/Raptor	Blocked by *bec1* RNAi	GFP::LGG1 puncta used to measure autophagy increase in *daf15/Raptor* RNAi worms	Hansen et al., 2008
	*Drosophila melanogaster*	+∼13% (median lifespan) in muscle overexpressing 4E-BP	N.D.	FOXO/4E-BP regulate proteostasis via autophagy	Demontis and Perrimon, 2010
AMPK	*Drosophila melanogaster*	Lifespan extension upon adult neuronal AMPK overexpression	Blocked by *Atg1* RNAi	Overexpression of Atg1 in adult neurons using elavGS driver extends lifespan and increases autophagy in the brain and the gut. Decreased insulin signalling associates with lifespan extension	Ulgherait et al., 2014
Dietary restriction	*Saccharomyces cerevisiae*	+∼100% (mean chronological lifespan)	Chronological lifespan extension blocked in Δ*atgl*,Δ*atg2*,Δ*atg7*, and Δ*atg8* strains	Autophagy promotes mitochondrial respiration under dietary restriction in chronological lifespan	Aris et al., 2013
	*Caenorhabditis elegans*	+∼21% (mean lifespan in *eat2* mutant)	Lifespan extension is dependent on *vps34, bed, unc51*, *and atg7*	Lifespan extension is dependent on the transcription factor PHA4/FOXA. Nuclear receptor NHR62 regulates DR-induced autophagy	Jia and Levine, 2007; Hansen et al., 2008; Toth et al., 2008; Heestand et al., 2013; Gelino et al., 2016
IIS	*Caenorhabditis elegans*	+∼100% (mean lifespan) in *daf2*	Lifespan extension dependent on *bec1*/Beclin and *atg18*	Autophagy increase independent on *daf16/FOXO*, but daf16/FOXO is required for lifespan extension of *daf2* mutants	Melendez et al., 2003; Hansen et al., 2008; Toth et al., 2008

*N.D., not determined.*

Another major autophagy regulator is mTORC1 (mTOR complex 1), which under nutrient-rich conditions, promotes growth and inhibits autophagy. This occurs through inhibitory phosphorylation of ULK1, and of the PIK3C3/VPS34 kinase complex, as well as by phosphorylation of TFEB (the lysosomal biogenesis transcription factor), leading to its cytosolic localization and block in transcriptional activity (Kim and Guan, 2019). Moderate inhibition of the mTOR pathway and its downstream effectors, such as S6K, either genetically or pharmacologically by rapamycin, is one of the most well studied anti-aging interventions. However, not all of its effects are mediated exclusively by increased autophagy, and lower protein synthesis is another pro-longevity effect of decreased mTOR signaling (Bjedov et al., 2010; Castillo-Quan et al., 2015; Saxton and Sabatini, 2017; Hansen et al., 2018).

In addition to amino acid sensing, saturated and unsaturated free fatty acids have also been shown to activate the autophagy process. This is most likely because circulating free fatty acids are abundant during starvation, which is the major autophagy trigger (Papsdorf and Brunet, 2019). Supplementation of food with ω-3 PUFA arachidonic acid (AA) or dihomo-γ-linolenic acid (DGLA) induces autophagy and extends lifespan in *Caenorhabditis elegans* (O’Rourke et al., 2013; Bustos and Partridge, 2017). In addition, α-linolenic acid induces autophagy in mammalian cells (O’Rourke et al., 2013). There is currently an increasing interest in the role of lipids in aging. Lipids that extend lifespan are mono-unsaturated fatty acids (MUFAs), such as oleic acid, *cis*-vaccenic acid and palmitoleic acid; albeit the longevity mechanism is linked specifically to modifiers of trimethylated lysine 4 on histone H3 (H3K4me3). This demonstrates an interesting link between lipids and chromatin (Han et al., 2017). However, the specific contribution of autophagy induction to the effects of these lipids is still unknown. Another important anti-aging observation is that in germline-less *glp-1* mutant worms, lacking the human ortholog of NOTCH1, longevity is mediated through the intricate interaction of increased lysosomal lipase LIPL-4 and up-regulation of autophagy (Wang et al., 2008; Lapierre et al., 2011; Hansen et al., 2018). LIPL-4 lipase liberates a MUFA, oleoylethanolamide, which mediates longevity effects by binding to lysosomal lipid chaperone LBP-8. This in turn leads to its nuclear localization and activation of the nuclear hormone receptors NHR-80 and NHR-49 (Folick et al., 2015). Taken together, these findings draw attention to the connection between autophagy, lipids and aging. They also demonstrate that autophagy can be regulated by certain fatty acid species, and vice versa. Indeed, active autophagy can alter cellular lipid composition through the activity of lysosomal lipases (Bustos and Partridge, 2017; Papsdorf and Brunet, 2019). Because autophagy is a degradative process, it must occur in moderation and in a coordinated manner. For instance, excessive lipophagy can liberate too many fatty acids from membrane degradation, which if not stored in lipid droplets, can lead to acylcarnitine accumulation and mitochondrial uncoupling and dysfunction (Nguyen and Olzmann, 2017). In addition, lipid alterations can also affect autophagic vesicular fusion (Koga et al., 2010). Clearly, more research on the interaction between autophagy, various lipid species, and aging is required. Particularly because of the pleiotropic role of lipids, this interaction can impact energy storage, signalling and transcription, as well as membrane composition and fluidity. Increasing proportions of unsaturated lipids in the membrane correlate with enhanced fluidity, but also increase the risk of lipid peroxidation, which is associated with reduced lifespan in worms and in several mammals (Shmookler Reis et al., 2011; Jove et al., 2013).

Overall, there is a clear link between energy sensors, nutrients, and nutrient signaling pathways and autophagy. Conversely, autophagy can influence nutrient signaling through liberation of degraded molecules, thus influencing cellular health status (Lahiri et al., 2019). Given that dietary restriction and modulation of nutrient signaling pathways are major pro-longevity interventions (Fontana et al., 2010; Fontana and Partridge, 2015), understanding how autophagy can be modified specifically to alter the metabolic profile of an individual for anti-aging purposes is an essential goal.

## Transcriptional Control of Autophagy and Aging

Several transcription factors are regulators of autophagy, TFEB (transcription factor EB) being one of the principal ones responsible for the coordination and expression of autophagy genes and lysosomal hydrolases. Overexpression of the TFEB ortholog HLH-30 was shown to increase lifespan in worms, with the extension being dependent on its nuclear localization (Lapierre et al., 2013). Another key transcription factor that regulates autophagy is FOXO (forkhead box class O), which is implicated in nearly all anti-aging interventions (Kenyon, 2010; Tullet, 2015; Martins et al., 2016). It hence became a prime pharmacological anti-aging target (Cheng, 2019). When unphosphorylated in the nucleus, FOXO activates the transcription of several autophagy genes (Mammucari et al., 2007; Zhao et al., 2007). Conversely, when phosphorylated in the cytoplasm it interacts with autophagy proteins (Cheng, 2019). In addition, autophagy directly and indirectly mediates FOXO degradation, providing a negative feedback loop, limiting dangerous excessive autophagic degradation (Webb and Brunet, 2014; Fitzwalter et al., 2018; Cheng, 2019). Interestingly, the FOXO3A variant is one of the few genes associated with longevity in human centenarians (Flachsbart et al., 2009). However, the precise biological effects associated with these FOXO variants are unknown. Another interesting transcriptional regulator of autophagy is p53. When in the cytosol, p53 promotes autophagy suppression, while in the nucleus it induces transcription of autophagy genes (Yee et al., 2009; Kenzelmann Broz et al., 2013). This tumor suppressor gene is one of the most commonly mutated cancer genes and can protect against cancer in mice when carefully overexpressed under endogenous control in a p53 overexpressor strain called “super p53” (Garcia-Cao et al., 2002). It also extends lifespan if combined with Ink4/Arf overexpression (Matheu et al., 2007). The effect of this “super p53” is in stark contrast to overexpression of constitutively active p53 under a heterologous promoter. This triggers excessive apoptosis, and although protecting mice from cancer, it accelerates the aging phenotype, most likely owing to depletion of the stem cell pools (Tyner et al., 2002; Finkel et al., 2007).

Given that autophagy is induced by these very different transcription factors strongly suggests that autophagy is a critical tool for dealing with most forms of stress. However, any excessive autophagy may be detrimental and cause cell death, given that it is a degradative process requiring moderate induction (Kang et al., 2007; Scott et al., 2007).

## Epigenetic Control of Autophagy and Aging

As described earlier, autophagy is initially triggered by post-translational protein modifications in the cytosol, and can also be regulated at the transcriptional level by various transcription factors. However, it can additionally be regulated at the epigenetic level (Fullgrabe et al., 2014; Lapierre et al., 2015; Baek and Kim, 2017). It is proposed that under prolonged autophagy induction, transcription factors help sustain autophagic flux, while further prolongation entails epigenetic changes that ensure that autophagy does not become lethal (Fullgrabe et al., 2013, 2014).

In addition to potentially preventing autophagy from going awry, it was proposed that an epigenetic regulation of the autophagic process could perhaps also lead to a memory effect enabling a quicker response to subsequent starvation events (Fullgrabe et al., 2014). “Memory effects” for longevity, transmitted across three generations, have been shown in *C. elegans* having lower H3K4me3 activity, owing to mutations in the corresponding methylation complex. Transgenerational inheritance is not a common feature of long-lived mutants and it does not occur in long-lived *daf-2* mutant worms, which have decreased IIS pathway owing to a mutation in the insulin receptor homolog *daf-2* (Greer et al., 2011). There is an association between autophagy induction and downregulation of H3K4me3 and H4K16ac (Fullgrabe et al., 2013). Increased autophagy is often linked to deacetylation, particularly of H4K16, which is mediated by Sirt1, and accompanied by the transcription of autophagy genes (Fullgrabe et al., 2014). Overexpression of Sirt1 is a well-studied putative anti-aging intervention (Lopez-Otin et al., 2013), again highlighting the intriguing link between autophagy and longevity. In general, different autophagy stimuli lead to deacetylation of H4K16 (Baek and Kim, 2017). Also LC3/Atg8 is deacetylated by Sirt1 and translocated to the cytosol upon autophagy initiation (Huang et al., 2015).

Another epigenetic alteration linked to autophagy is H3R17, resulting from the coactivation of TFEB by CARM1 (Shin et al., 2016). Autophagy can be suppressed by some of the common epigenetic changes such as G9a-mediated H3K9 dimethylation (Artal-Martinez de Narvajas et al., 2013) and silencing histone mark H3K27 trimethylation via EZH2 (Enhancer of Zest Homologue2) (Wei et al., 2015). Interestingly, H3K27 is linked to aging, albeit evolutionary conservation of this remains to be confirmed. Loss of chromatin repression was observed during aging in species from *C. elegans* to humans (Bennett-Baker et al., 2003). However, while an increase in H3K27me3 by RNAi against UTX-1 demethylase extends lifespan in worms (Jin et al., 2011; Maures et al., 2011), it is a decrease in H3K27me3 in flies by mutations in PRC2 components E(z) and ESC, that promotes longevity (Siebold et al., 2010). Thus, the intriguing relationship between autophagy and epigenetics has the potential to provide invaluable insights into the aging process itself.

Collectively, there are numerous genetic and epigenetic autophagy regulators, most of which have already been implicated in aging. Targeting these offers numerous possibilities for modulating autophagy to improve health in older age. However, many of these autophagy regulators have pleiotropic effects, implying that any potential treatments need to be carefully evaluated.

## Autophagy as a Common Denominator of Anti-Aging Interventions

Aging is accompanied by progressive decline of autophagy in many organisms (Hansen et al., 2018). A reduction in autophagy during aging was demonstrated in a study that carefully examined autophagy in different tissues throughout adulthood of long-lived *daf-2* and *glp-1 C. elegans* mutants, and showed that intestinal autophagy inhibition abolishes longevity only in *glp-1* mutants (Chang et al., 2017). Mice deficient for essential autophagy genes, such as *atg5, atg7* or *atg12*, are neonatal lethal and die within 1 day due to failure to adapt to starvation and from a suckling defect (Kuma et al., 2017). Moreover, this lethality can be rescued by the neuron-specific expression of ATG5 (Yoshii et al., 2016). Neuronal and glial specific deletion of either *atg5* or *atg7* results in viable and short-lived mice displaying neuronal protein accumulation and neurodegeneration (Hara et al., 2006; Komatsu et al., 2006). This highlights the importance of autophagy in removing damaged proteins in non-dividing neuronal tissue, and the potential of therapeutic autophagy enhancement in neurodegenerative disease (Menzies et al., 2017; Scrivo et al., 2018).

Evidence for the role of autophagy in aging was first shown in *daf-2* long-lived worms, where RNAi-mediated downregulation of the autophagy gene *bec-1* completely abrogated their pronounced longevity (Melendez et al., 2003). Since this discovery, dependence on autophagy enhancement has been demonstrated in nearly all longevity-promoting interventions. For instance, lifespan extension by dietary restriction, mTOR inhibition, AMPK up-regulation, mitochondrial mutations, and the above mentioned germline *glp-1* mutation, all require functional autophagy for lifespan extension (Toth et al., 2008; Hansen et al., 2018). In all these long-lived mutants, lessening autophagy by RNAi returns lifespan toward wild type levels. However, controls treated with similar autophagy-reducing RNAi interventions do not display altered longevity, suggesting that the residual autophagy levels are sufficient to maintain normal lifespan. It is worth noting that the nutrient-sensing pathways implicated in longevity have pleiotropic effects on metabolism, and often, under conditions when autophagy is up-regulated, this also impacts on other anti-aging processes such translation. It is thus challenging to fully evaluate exact contributions of different down-stream effectors on overall longevity. Another intriguing finding is that if autophagy is inhibited in neurons of post-reproductive adult worms, by targeting genes involved in the early stage of the autophagy process such as *bec-1*, this results in an extension in lifespan (Wilhelm et al., 2017). One plausible explanation for this is that if aged worms have impaired autophagy flux in the neuronal tissues, then inhibiting autophagy initiation stops clogging the system even further, preventing neuronal decline (Wilhelm and Richly, 2018). Another example where inhibiting autophagy is beneficial is in the intestine of the worm, where this approach limits conversion of intestinal biomass to yolk, thereby preventing age-related visceral pathologies (Ezcurra et al., 2018). This suggests that, rather than being a result of age-associated “wear and tear,” some pathologies may result from a precise genetically-driven biological program (Ezcurra et al., 2018).

This detrimental effect of autophagy late in life has only been observed in worms. It remains to be investigated if autophagy behaves similarly as an antagonistic pleiotropic process in mammals, being beneficial in the young, but detrimental in the aged organism. In this case, interventions to enhance autophagy to improve healthspan may need to be initiated early to prevent tissue deterioration, while later in life once the host’s autophagic machinery has already begun to fail, it should be carefully considered whether autophagy needs to be enhanced or inhibited. However, it should be noted that in mice, the autophagy activating drug rapamycin is beneficial once administered later in life (Harrison et al., 2009), suggesting that either in mammals autophagy activation is beneficial irrespective of age, unlike in worms (Wilhelm et al., 2017; Wilhelm and Richly, 2018), or that rapamycin-mediated downregulation of translation is an important effect for healthspan benefits in the aged mice.

In order to improve health during aging, understanding those alterations that increase lifespan are essential, as they directly demonstrate the importance of a given paradigm in aging. Manipulations that increase autophagy directly are valuable but sparse, and complicated by the fact that that numerous autophagy genes are involved in different stages of this multistep process. Moreover, overexpression of only one autophagy gene does not necessarily trigger autophagy. Nevertheless, there are some very valuable exceptions that directly show how important this process is in aging (Hansen et al., 2018; Maruzs et al., 2019). For instance, overexpression of Atg8a in neurons (Simonsen et al., 2008), as well as Atg1 overexpression in neuronal tissue (Ulgherait et al., 2014) or muscle (Bai et al., 2013), extends lifespan in *Drosophila*. In addition, mammalian lifespan was extended by an ubiquitous increase of Atg5 in mice, and was accompanied by improved motor function (Pyo et al., 2013). Moreover, knock-in of *Becn1* bearing the Phe121Ala mutation, which interrupts its interaction with its negative regulator BCL2, leads to increased autophagy flux and longevity in both male and female mice (Fernandez et al., 2018). These observations are very important to elucidate how an upregulation in autophagy leads directly to benefits at the organismal level. Further studies of autophagy manipulation in different tissues will help to elucidate further tissue-specific effects and the impact of these on organismal aging (Hansen et al., 2018). In particular, combining longevity experiments with healthspan parameters, such as motor function, cardiovascular deterioration, neuronal loss and insulin sensitivity, will facilitate the discovery of pharmacological targets for disease prevention and treatment.

## Autophagy – a Boost for Proteostasis

The role of autophagy in delaying aging is commonly attributed to its capacity to degrade damaged proteins and contribute to cellular proteostasis (Hansen et al., 2018). Loss of proteostasis is a prominent feature of aging and a major risk-factor for age-related neurodegenerative disorders such as Alzheimer’s and Parkinson’s diseases (Lopez-Otin et al., 2013; Kaushik and Cuervo, 2015; Sands et al., 2017; Hipp et al., 2019). A recent detailed proteomic profiling in worms revealed that a third of the proteome changes in abundance by at least two or threefold with aging (Walther et al., 2015). Interestingly, long-lived *daf-2* mutant worms have increased proteasome subunits, but not autophagy proteins, and they maintain proteostasis by sequestering problematic proteins into less toxic chaperone-containing aggregates (Walther et al., 2015). This is in accordance with findings in the neurodegeneration field suggesting that, if unfolded proteins cannot be re-folded, or degraded, then the resulting unfolded soluble oligomeric proteins are far more toxic to the cell than insoluble aggregates (Cohen et al., 2006; Hipp et al., 2019). Consistent with this, reduced insulin signaling in long-lived insulin-like growth factor 1 (IGF-1) heterozygous mice are protected against amyloid β-peptide (Aβ) proteotoxicity by favoring a shift from toxic oligomers toward less toxic aggregates (Cohen et al., 2009). It should be noted, however, that the relationship between insulin signaling and neurodegenerative disease is very complex and not well understood (Ribe and Lovestone, 2016).

Proteostastic mechanisms consist of protein synthesis, protein folding and protein degradation, all of which are interconnected in order to achieve a balanced proteome and robust stress responses when required (Kaushik and Cuervo, 2015; Hipp et al., 2019). The protein synthesis pathway, mTOR, has a well described effect on autophagy inhibition, and has recently also been linked to the proteasome. It has been shown to both activate it via the nuclear factor erythroid 2-related factor 1 (Nrf1) to balance active translation (Zhang et al., 2014), and inhibit the proteasome in a different experimental set up (Zhao et al., 2015). mTOR inhibition can relieve proteotoxic stress by decreasing translation, and specifically, by allowing translation of additional chaperones (Su et al., 2016). Decreasing translation through mutations in mTOR pathway genes, such as S6K (Selman et al., 2009), or by or overexpression of the mTOR suppressor TSC1/2 (Kapahi et al., 2004), are well-described anti-aging interventions for which several underlying mechanisms are suggested. For example, reduced translation extends longevity by reducing the protein load for the proteostatic machinery. Furthermore, the reduction in energy expenditure associated with reduced translation can be invested into cell maintenance processes. Moreover, differential translation may lead to stress resilience and delayed aging (Mehta et al., 2010; Steffen and Dillin, 2016). In addition, it appears that enhancement of proteostasis, either by improved protein folding or by degradation, is also a potentially successful anti-aging strategy (Lopez-Otin et al., 2013). For example, overexpression of the heat shock factor HSF-1 has repeatedly been shown to extend lifespan in worms (Hsu et al., 2003; Li J. et al., 2017). Additionally, overexpression of the proteasomal subunit Rpn6, which stabilizes the 20S core particle and 19S regulatory particle, can induce proteasome activity and extend lifespan in *C. elegans*. This is an exciting finding as the proteasome has numerous subunits and this suggests that targeting only one key subunit is sufficient to intensify proteasomal degradation (Vilchez et al., 2012). In *Drosophila*, overexpressing the β5 subunit of the 20S in the whole organism (Nguyen et al., 2019), or just in neurons, extends lifespan (Munkacsy et al., 2019). While whole-body β5 subunit overexpression increases proteostasis in muscle (Nguyen et al., 2019), neuron-specific overexpression prevents age-related decline in learning and memory (Munkacsy et al., 2019). Enhancement of autophagy by overexpression of FOXO transcription factor in muscle tissues in *Drosophila* is beneficial as well (Demontis and Perrimon, 2010). This tissue-specific upregulation of autophagy in muscle tissues mediates a reduction in aggregate formation during aging, with an improvement in proteostasis in the whole organism, as well as increased longevity (Demontis and Perrimon, 2010).

Autophagy function is highly complementary to that of the proteasome, which targets small short-lived proteins. Autophagy degrades large and long-lived proteins, protein aggregates, entire defective organelles, and essentially any cellular material that is too bulky for proteasomal degradation (Dikic, 2017; Kocaturk and Gozuacik, 2018). Interestingly, results in mammalian cells suggest that there is significant crosstalk and a degree of compensation between these two degradative processes. If the proteasome is inhibited, then autophagy enhances to compensate (Kocaturk and Gozuacik, 2018). However, there is less evidence to support a role for the proteasome in compensating for a block in autophagy. This is likely a result of the fact that the proteasome cannot accomplish all autophagic functions and cannot degrade organelles (Dikic, 2017; Kirkin, 2019). A common characteristic of these two degradative pathways is that they both recognize ubiquitinated substrates. Autophagy often degrades aggregates linked to K63-based polyubiquitin chains, while substrates for the proteasome mainly have K48-polyubiquitin chains (Kocaturk and Gozuacik, 2018). However, it should be noted that the specificity of autophagy toward K63 ubiquitinated substrates is not that clear. For example, in autophagy-deficient mice all types of ubiquitin chains accumulate (Riley et al., 2010).

## Autophagy – Cytoplasmic Influence on DNA Damage

Aging is accompanied by accumulation of damage and damaged organelles, among which damaged DNA is the most strongly linked to aging. Various syndromes originating from deficiency in DNA repair enzymes recapitulate some of the aging phenotypes and are models of accelerated aging. Examples include Werner and Bloom syndrome, which are caused by mutations in the WRN and BLM genes, respectively, both of which are RecQ-like helicases (Carrero et al., 2016). Cockayne syndrome is another progeria model deficient in ERCC6 or ERCC8, while Hutchinson-Gilford progeria bears a mutation in laminin A gene (LMNA), whose product is a key structural component of nuclear lamina. In addition, defective DNA repair associated with mutations in genes such as Ku70, Ku80, Ercc1, and Xpd leads to shorter lifespan in mice (Vermeij et al., 2016). This strongly suggests a role for DNA damage in aging (Vermeij et al., 2016).

Interestingly, autophagy affects DNA repair, a finding that came as a surprise given that autophagy is mainly a cytoplasmic process. Initially it was reported that mammalian cells lacking *beclin1* or *atg5* genes have increased genomic instability (Karantza-Wadsworth et al., 2007; Mathew et al., 2007). Since, autophagy was linked to different components of the DNA Damage Response (DDR), as we discuss below, this strengthens the impact of autophagy on genomic integrity and thus aging (Eliopoulos et al., 2016; Hewitt and Korolchuk, 2017).

Maintenance of genomic stability is key for survival. Therefore a complex DDR, with the ability to detect various types of DNA aberrations and engage appropriate DNA repair systems, has evolved (Ciccia and Elledge, 2010). DNA lesions, through post-translational histone modifications, trigger relaxation of the chromatin, and inhibit replication and transcription, in order to promote DNA repair instead (Jeggo et al., 2017). For instance, removal of HP1α (heterochromatin protein 1 alpha) from DSB sites allows formation of Rad51 nucleoprotein filaments and successful homologous recombination (HR). Interestingly, HP1α is ubiquitinated by RAD6, which then triggers autophagic degradation of HP1α and loosening of chromatin compaction for successful double strand break (DSB) repair in mammalian cells (Chen et al., 2015). DSBs are bound by MRN (Mre11-Rad50-Nbs1) and then ATM (ataxia-telangiectasia mutated) kinase, which becomes autophosphorylated and activated, ultimately leading to either homologous recombination (HR) repair, that can occur exclusively in G2/S phase of the cell cycle, or non-homologous end joining (NHEJ). The latter functions in all phases of the cell cycle (Ciccia and Elledge, 2010). Although NHEJ is traditionally considered more erroneous than HR, it has been shown that the increased DNA resection required in different types of NHEJ and HR correlates well with the level of mutations (Rodgers and McVey, 2016). For instance, HR can be mutagenic when hyper-resection by Rad52 mediates single-strand annealing-type of HR (Ochs et al., 2016). Single strand breaks are recognized by RPA (replication protein A), which recruits ATR (ataxia-telangiectasia and Rad3 related) kinase, promoting phosphorylation of p53 and Chk1 as part of the irradiation response. In both ATM-Chk2 and ATR-Chk1 axes of repair, among which cross talk exists, one of the key initial events for DDR is phosphorylation of γH2AX by ATM, ATR or DNA-PKcs (Ciccia and Elledge, 2010; Blackford and Jackson, 2017). In addition to DSB and single strand break (SSB) repair, base excision repair (BER), and nucleotide excision repair (NER) are scanning DNA for different types of damage. Mismatches occurring during replication are fixed by mismatch repair (MMR), while specialized translesion synthesis (TLS) polymerases tolerate damage and avoid replication fork stalling (Ciccia and Elledge, 2010; Jeggo et al., 2017). As well as these DNA damage sensors, mediators and effectors, a number of other cellular responses are essential when timely DNA repair is not achieved (Ciccia and Elledge, 2010). These include cell cycle arrest, changes in energy and metabolism and initiation of cell death. Interestingly, autophagic cell death in cells undergoing a replicative crisis is critical for eliminating cells with genomic instability (Nassour et al., 2019).

There is an increasing number of conceptually interesting links between autophagy and DDR (Eliopoulos et al., 2016; Hewitt and Korolchuk, 2017). For instance, valproic acid, which is an autophagy stimulator and histone deacetylase inhibitor, targeting HDACs Hda1 and Rpd3, limits DDR by stimulating autophagic degradation of acetylated recombination protein Sea2/CtIP. This confers DNA damage sensitivity – an interesting finding linking acetylation, autophagy and DSB repair (Robert et al., 2011). Genomic instability of autophagy-deficient cells (Karantza-Wadsworth et al., 2007; Mathew et al., 2007) is linked to compensatory upregulation of proteasomal degradation, resulting in less phospho-Chk1 in response to damage. Consequently HR is impaired, making these cells more reliant on NHEJ (Liu et al., 2015). Chk1 is also a substrate for CMA, and high levels of Chk1 accumulating in the nucleus are equally detrimental to genome integrity, as shown when CMA is inhibited and the MRN complex is destabilized (Park et al., 2015). This highlights the fact that in DNA repair, alterations in enzyme activity need to be subtle, and possibly coordinated with other interacting proteins, otherwise repair outcome is perturbed. Furthermore, it has been shown in the hematopoietic system that increasing autophagy using rapamycin is protective against radiation and induced HR and NHEJ (Lin et al., 2015). Rapamycin-independent autophagy is also described in yeast, where DNA damaging agents, acting through DDR kinases Mec1/ATR, Tel1/ATM, and Rad53/CHEK2, induce a novel type of autophagy, named genotoxin-induced targeted autophagy, which relies on Atg11 (Eapen et al., 2017). Another interesting DNA repair link involves the p62/SQSTM1 autophagy receptor protein, which plays a role in proteasomal degradation of ubiquitinated proteins (Liu et al., 2016). p62/SQSTM1-dependent proteasomal degradation of recombination proteins filamin A (FLNA) and RAD51 in the nucleus is excessive under high levels of P62/SQSTM1. This occurs when the autophagic degradation of p62 is impaired, leading to defective DNA repair (Hewitt et al., 2016). A nuclear increase of p62 in autophagy-deficient mammalian cells also inhibits the E3 ligase RNF168, leading to deficient H2A ubiquitination, thereby thwarting recruitment of both NHEJ and HR DNA repair enzymes to the damaged sites (Wang et al., 2016). Inefficient p62-mediated degradation of GATA4 transcription factor leads to induction of cellular senescence, linking autophagy and senescence (Kang et al., 2015). In summary, these findings demonstrate intriguing connections between DNA and protein damage control, and highlight the effect of low levels of nuclear p62 for preventing genomic instability (Mathew et al., 2009). Above, we focused on the links between DSB and SSB repair and the autophagy process. However, BER, NER, and MMR are also associated with autophagy, albeit to a lesser extent. Indeed, it was recently reported that DNA damage, induced by 5-FU, is accompanied by BER and MMR activation and repair, resulting in autophagy-mediated cell death (SenGupta et al., 2013). In the presence of a different cytotoxic chemical, 6-TG, MMR induces autophagy via p53 (Zeng et al., 2007). Finally, in addition to the above mentioned DDR proteins that are modulated by the autophagic status of the cell, autophagy can result in the degradation of entire micronuclei, which are chromosomal fragments that are not incorporated into daughter cells, and which are common markers of genotoxicity (Rello-Varona et al., 2012).

In conclusion, despite being a cytoplasmic process, autophagy can affect numerous aspects of DNA repair. Moderate autophagy enhancement appears to mainly exert positive effects on DDR. It would therefore be interesting to examine whether lifespan in long-lived autophagy mutants depends on DDR. Owing to the complexity of DNA repair systems, overexpression of a single enzyme does not necessarily enhance the entire type of repair, similar to autophagy, making lifespan extension by genetic up-regulation of DDR difficult to investigate. However, these types of findings are particularly valuable for understanding aging, and a few examples do exist. A large overexpression screen in *Drosophila* uncovered that ubiquitous enhancement of mei-9/XPF resulted in a consistent increase in longevity among tested candidates genes (Shaposhnikov et al., 2015). Remarkably, an extensive study of 18 rodent species revealed that DSB repair co-evolves with longevity, not NER; the latter correlating with sunlight exposure of different species, not their lifespan (Tian et al., 2019). More specifically, it is five amino acids in SIRT6 that dictate the different activities of both HR and NHEJ-types of DSB repair, and which are accountable for longevity (Tian et al., 2019). Thus, it would be interesting to develop drugs that enhance DNA repair, without inflicting DNA damage, as they may prove to be effective anti-aging drugs.

## Degrading Cellular Organelles – Mitophagy and Aging

The precise role of mitochondria in aging is an interesting and heavily debated topic. It is increasingly becoming apparent that their degradation by mitophagy, whereby mitochondrial number is regulated and damaged mitochondria recycled, is important for aging and disease. Mitochondrial fission and fusion are vital for the maintenance of mitochondrial shape and health, and are regulated by MFN1/2, FIS1, DRP1, and OPA1. For instance, under starvation conditions mitochondria are protected from mitophagy; mitochondrial fusion results in mitochondria predominantly in the elongated form, thwarting their degradation (Gomes et al., 2011). On the other hand, it is key that damaged parts of the mitochondria are separated through a fission process. These non-functional mitochondria are then degraded by ubiquitin-dependent pathways. Depolarization of the mitochondrial membrane leads to accumulation of PINK1 on the outer membrane, where it phosphorylates ubiquitin and the E3 ubiquitin ligase PARKIN, leading to activation of PARKIN and the ubiquitination of mitochondrial membrane proteins (Kane et al., 2014; Koyano et al., 2014; Gladkova et al., 2018; Harper et al., 2018; Palikaras et al., 2018). It is thought that selective autophagy adaptors, NDP52 and TAX1BP1 bind to ubiquitinated mitochondria, enabling their degradation by selective autophagy (Lazarou et al., 2015). A recent paradigm shift has shown that for various forms of selective autophagy, the autophagy adaptors lead to recruitment of the autophagy initiation machinery, initiating a cascade of autophagosome formation locally (Ravenhill et al., 2019; Vargas et al., 2019; Zachari et al., 2019). Additionally, in ubiquitin-independent mitophagy, mitochondria are linked to the autophagosomes by BNIP3, FUNDC1 and NIX, which are specific autophagy receptors. Enhancing mitophagy has emerged as a promising therapeutic strategy in Parkinson’s disease and other age-related disorders (Padmanabhan et al., 2019).

Mitochondria malfunction with age, leading to perturbations in metabolic and energy homeostasis, releasing reactive oxygen species (ROS) (Lopez-Otin et al., 2013; Munkacsy and Rea, 2014). Increases in ROS, causing molecular damage, have long been considered important culprits in aging. However, careful reassessment suggests that mutants displaying subtle increases in ROS can be long-lived, while highly elevated ROS levels are detrimental (Lopez-Otin et al., 2013; Ristow and Schmeisser, 2014). This is a hormesis effect, where low dose of a substance has a positive stimulatory effect but, contrarily, high dose is toxic (Gems and Partridge, 2008). ROS can be protective until a certain threshold of damage is reached, owing to their role in signaling and ability to trigger defense responses that result in increased robustness and longevity. If this threshold is surpassed, then the amount of DNA damage that occurs becomes toxic for the organism and lifespan is shortened (Gems and Partridge, 2008; Ristow and Schmeisser, 2014; Schieber and Chandel, 2014).

Unexpectedly, given the essential role of mitochondria in physiological functions, it has been observed that mutations in some mitochondrial subunits extend lifespan in worms (Feng et al., 2001; Dillin et al., 2002), flies (Copeland et al., 2009) and mice (Liu et al., 2005). Mutations in all mitochondrial respiratory chain complex genes, except complex II, can extend lifespan in *C. elegans* (Munkacsy and Rea, 2014). It was suggested that this effect occurs as a result of an imbalance between nuclear and mitochondrial encoded subunits, inducing the mitochondrial unfolded protein (UPR^mt^) response. Furthermore, it is thought that this does not occur in complex II mutants, since it is the only complex that is exclusively encoded by the nuclear genome (Houtkooper et al., 2013). Remarkably, the triggering of the UPR^mt^ in one tissue can be communicated to another tissue, and this communication leads to enhanced protection against organismal stress (Durieux et al., 2011; Zhang et al., 2018). To fully explain the increased longevity of mitochondrial mutants, in addition to induction of the UPR^mt^, it was proposed that these mutants display enhanced mitophagy, removing defective mitochondria and improving cellular homeostasis. Indeed, an imbalance between mitophagy and mitochondrial biogenesis plays an important role in aging in *C. elegans* (Palikaras et al., 2015). Also, increasing mitochondrial fission by overexpression of Drp1 in *Drosophila* enhances mitophagy, maintains mitochondrial respiratory function during aging and extends healthspan (Rana et al., 2017). Another interesting observation linking autophagy and mitochondria is that mitochondrial permeability determines the effect of autophagy on lifespan (Zhou et al., 2019). More precisely, low mitochondrial permeability is required for various autophagy-mediated lifespan extension effects, while increased permeability is detrimental to the organism (Zhou et al., 2019). In addition, there is evidence to support ROS as secondary messengers, not only as damaging agents. For example, mitochondrial ROS produced by reversing electron transport leads to lifespan extension in *Drosophila* (Scialò et al., 2016). Overall, it seems that mild induction of stress responses, when damage appears to not be overwhelming, is beneficial for aging, possibly by inducing defense mechanisms and preparing the cell for any subsequent damaging insults that may incur. However, as soon as the stress threshold is exceeded, then either excessive damage, or the stress-response pathway itself, may become detrimental and lead to life-shortening consequences.

## Drugs Targeting Autophagy for Better Health During Aging

One of the critical challenges in modern societies is to improve health during aging. The steep increase in life expectancy seen in populations over recent decades is recognized as a remarkable accomplishment due to advancements in medicine, public health and technology. However, this has been accompanied by a greater number of individuals suffering from age-related diseases, such as cancer and neurodegenerative disorders. Enhanced autophagy is a common mechanism among putative anti-aging interventions, and a number of such drugs exist that are capable of increasing the autophagic process (Table 2). This in turn opens up promising clinical avenues to improve health in the elderly, and to prevent the onset of diseases of old age through pharmacologically-modulated autophagy (Partridge et al., 2018; Campisi et al., 2019; Singh et al., 2019).

**TABLE 2:** Compounds that increase autophagy with potential anti-ageing properties.

**Compound**	**Organism**	**Longevity**	**Blocked by autophagy impairment?**	**Non-autophagic mechanism**	**References**
Rapamycin	*Caenorhabditis elegans*	+19% (mean lifespan)	N.D.	Requires an intact SKN-1/Nrf transcription factor	Robida-Stubbs et al., 2012
	*Drosophila melanogaster*	+15% (median lifespan)	Lifespan extension blocked by atg-5 RNAi	Reduces translation and lifespan and also blocked by overexpression of constitutively active ds6k/S6K	Bjedov et al., 2010
	Mice	Lifespan extension when started either early or late in life. An optimal dose with maximal lifespan extension has not been determined	N.D.	Reduced S6K phosphorylation is used as readout of mTORC1 inhibition	Harrison et al., 2009; Miller et al., 2014
Torin 1	*Drosophila melanogaster*	+60% (median lifespan) of short lived controls	N.D. Autophagy activation determined by lipidated Atg8/LC3	N.D.	Mason et al., 2018
Trehalose	*Caenorhabditis elegans*	+32% (mean lifespan)	Lifespan extension is dependent on LGG1/Atg8/LC3 and Beclin	Lifespan extension is also dependent on the transcription factor DAF16/FOXO	Honda et al., 2010; Seo et al., 2018
Spermidine	*Saccharomyces cerevisiae*	Extends both chronological and replicative lifespan	Partial and condition-dependent on Atg7	N.D.	Eisenberg et al., 2009
	*Caenorhabditis elegans*	+15% (mean lifespan)	Lifespan extension dependent on *bec-1*/Beclin	N.D.	Eisenberg et al., 2009
	*Drosophila melanogaster*	+30% (mean lifespan)	Lifespan extension abolished in flies lacking *atg7*	N.D.	Eisenberg et al., 2009
Urolithin A	*Caenorhabditis elegans*	+45% (mean lifespan)	Lifespan extension is dependent on several genes involved in macroautophagy and mitophagy	Lifespan extension also dependent on *aak-*2/AMPK and *skn-*J/Nrf	Ryu et al., 2016
Valproic acid	*Caenorhabditis elegans*	+35% (mean lifespan)	N.D.	Increased DAF-16 nuclear localization	Evason et al., 2008
Lithium	*Schizosaccharomyces pombe*	+10% (median chronological lifespan)	N.D.	Reduced translation	Sofola-Adesakin et al., 2014
	*Caenorhabditis elegans*	+46% (median lifespan)	N.D.	Changes in chromatin structure and histone methylation	McColl et al., 2008
	*Drosophila melanogaster*	+16% (median lifespan)	No change in lipidated Atg8/LC3, not blocked by heterozygous loss of *atgl* and additive lifespan extension in combination with rapamycin	Increased activation of the redox and xenobiotic response by CncC/Nrf2	Castillo-Quan et al., 2016, 2019

*N.D., not determined.*

Autophagy has a complex relationship with cancer and neurodegeneration (Jiang and Mizushima, 2014; Leidal et al., 2018). Our understanding of the role of autophagy has improved significantly from the study of a variety of genetically engineered mouse models displaying a lack of autophagy, either from *Atg7* or *Atg5* deletion, in combination with an oncogene mutation Kras^G12D^, causing either pancreatic intraepithelial neoplasia (PANIN) (Rosenfeldt et al., 2013) or liver adenoma (Takamura et al., 2011). In accordance with the above-mentioned findings, the autophagy-deficient cells suffer from genomic instability (Karantza-Wadsworth et al., 2007; Mathew et al., 2007). Critically, these benign early stage tumors never progress into more malignant ones. This suggests that active autophagy is required for further tumor transformation, most likely to sustain tumor cell survival under stressful conditions owing to lack of nutrients and hypoxia (Kimmelman and White, 2017; Poillet-Perez and White, 2019). It thus appears that human cancers preserve autophagy function. This also points to the fact that complete autophagy ablation in mouse models, while providing a conceptually important insight into the role of autophagy in cancer, may not represent the pathological landscape of the majority of advanced malignant tumors in humans (Poillet-Perez and White, 2019).

Tumors require autophagy to provide the cell with key metabolic intermediates, such as nucleotides and TCA (tricarboxylic acid) cycle metabolites (Guo et al., 2016). This highlights an aspect of tumor vulnerability, whereby autophagy inhibition may be more harmful to tumor tissue than normal healthy tissue. It should be noted that autophagy in the surrounding tissue contributes to tumor growth. One of the key metabolites provided by the host that promotes tumor growth in the pancreas is arginine, which is degraded in autophagy-deficient mice by liver secreted arginase 1 (ARG1) (Poillet-Perez et al., 2018). However, not all tumors tested are arginine auxotrophs, and therefore sensitive to the autophagy status of the host. Another example of the role of autophagy in extra-tumoural tissue can be seen in stroma-associated pancreatic stellate cells, which support pancreatic ductal adenocarcinoma through autophagic alanine secretion (Sousa et al., 2016). Overall, although autophagy can have both tumor-promoting or -reducing effects, depending on the stage of the tumor and its genetic makeup, it is autophagy inhibitors such as hydroxychloroquine that have been primarily tested as anti-cancer therapies, as they are expected to hinder growth in advanced tumors (Poillet-Perez and White, 2019). However, many cancer drugs, such as rapamycin, are autophagy activators. Thus, careful evaluation of the role of autophagy in different tumor types is required. In particular, the effect of autophagy modulation in different drug combinations and regimes needs to be studied in the context of tumor growth.

In the case of neurodegenerative disease, drugs enhancing autophagy, rather than inhibiting, are the most studied. Most misfolded proteins that are deposited in the brains of people affected by neurodegenerative disorders, such as α-synuclein, tau and huntingtin, are autophagy substrates (Menzies et al., 2017). There is evidence that there is insufficient autophagy in many neurodegenerative disorders, and that enhanced autophagy may offer promising therapeutic benefits (Menzies et al., 2017). However, if during aging or in disease, the autophagy process is impaired at any stage, then further up-regulation of autophagy pharmacologically will not aid in aggregate clearance. In keeping with this, an *in vitro* study demonstrated that autophagy enhancement by rapamycin, or due to starvation, surprisingly can lead to increased toxicity (Tanik et al., 2013). We therefore need to fully understand the role of autophagy, and how perturbations in autophagy flux affect protein aggregation and clearance in neurodegenerative disease. It also highlights the need for more refined autophagy-modulating drugs that can target different stages of the autophagy process. When using autophagy altering drugs that affect other cellular processes, such as in the case of rapamycin, which down-regulates translation, then this additional effect of altered translation on the disease progression should also be evaluated. In aging, the differential rapamycin effects, including the inhibition of translation, and activation of autophagy, are beneficial for longevity (Bjedov et al., 2010), but this may not be the case in disease-related situations. Enhancement of autophagy flux thus offers a potential promising strategy for prevention of neurodegenerative disorders, by improving neuronal proteostasis and preventing cellular toxicity. However, different personalized approaches may be required in terms of optimizing the effects of autophagy modulation and minimizing negative pleiotropic effects of pharmacological interventions.

Similar to neurodegeneration, enhanced autophagy has proven to be an important pharmacological intervention in models of aging. Autophagy can be increased upon inhibition of growth pathways, which are also principal targets of cancer therapy. Interestingly, some of the anti-cancer drugs, such as rapamycin and the MEK/ERK inhibitor trametinib, have been shown to exert pro-longevity effects (Castillo-Quan et al., 2015). The very same nutrient-sensing/growth pathways that are highly up-regulated in cancer, enabling uncontrollable growth of tumor cells, can also promote health if mildly down-regulated to inhibit nutrient-sensing in normal cells (Bjedov and Partridge, 2011; Campisi et al., 2019). Anti-cancer drugs are used in very high concentrations for anti-cancer therapy, often causing side effects. However, in stark contrast, when administered in very low doses to normal non-transformed tissue, they promote anti-aging and disease preventative effects (Bjedov et al., 2010; Slack et al., 2015). It is important to note, however, that not all anti-cancer drugs in small concentrations are expected to improve health. Indeed, many are DNA-damaging agents, such as temozolomide and carboplatin, with negative effects on genome stability (Cheung-Ong et al., 2013). Moreover, as we refine the tools and screening procedures to monitor the autophagic process in healthy and diseased-tissue, it is likely that we will uncover many more autophagy enhancers and inhibitors (Pengo et al., 2018; Panda et al., 2019).

Autophagy activation can be achieved using two licensed drugs that are used to treat epilepsy and mood disorders, namely valproic acid and carbamazepine, as well as the mood-stabilizing drug lithium (Sarkar et al., 2005; Schiebler et al., 2015; Kerr et al., 2018). Valproic acid and lithium have proven anti-aging effects in model organisms. All of these drugs have been linked to mTOR-independent autophagy activation via reducing the recycling of inositol, which in turn reduces inositol 1,4,5-trisphosphate (IP_3_), disrupting the Beclin-Bcl-2 complex (Ravikumar et al., 2010). Indeed, mTOR-independent autophagy was recently shown to promote longevity and healthspan in mice (Fernandez et al., 2018). Valproic acid has been shown to extend lifespan and to reduce age-related locomotor decline in *C. elegans* (Evason et al., 2008). Interestingly, while a clear epistatic mechanism of action was not studied, it was observed that valproic acid induces DAF-16/FOXO nuclear accumulation, which may in turn regulate the transcription of autophagy genes (see above). Additionally, the combination of optimal concentrations of valproic acid and trimethadione, another anticonvulsant, had additive effects in extending worm lifespan (Evason et al., 2008). In mice, combinations of valproic acid and lithium retarded the onset and severity of symptomatology, and prolonged the lifespan of a mouse model of amyotrophic lateral sclerosis (ALS) (Feng et al., 2008). Although the main lithium trial in ALS patients was negative (Group et al., 2013), ALS is linked to several different gene mutations, and therefore analysis of genetically defined sub-groups of patients at earlier stages of disease may be necessary. Recent post-mortem analyses of the cerebral cortex transcriptome of ALS patients revealed at least three distinct molecular signatures in which patients could be classified. Intriguingly, the most common molecular subtype included aberrant expression of oxidative and proteostasis stress responses, which included autophagy-related genes (Tam et al., 2019). Therefore, in addition to genetic testing for common gene mutations, a better understanding of the molecular basis of ALS may lead to treatment stratification based upon disease molecular signatures (Group et al., 2013).

The lifespan extending effects of lithium were first shown in *C. elegans* (McColl et al., 2008) and have been confirmed in yeast and flies (Sofola-Adesakin et al., 2014; Castillo-Quan et al., 2016). In addition, increasing concentrations of lithium in the drinking water have been linked to a reduction in all-cause mortality in a Japanese population (Zarse et al., 2011). Interestingly, none of these studies have tied the longevity properties of lithium to increased autophagy. In yeast, the effects of lithium were linked to reduced translation (Sofola-Adesakin et al., 2014), in *C. elegans* it was associated with histone methylation and chromatin structure, while in *Drosophila* it was shown to inhibit glycogen synthase kinase-3 (GSK-3) activity and the transcriptional activation of the *cap’n’collar C (CncC)/Nrf2*. The latter regulates redox and xenobiotic metabolism (Castillo-Quan et al., 2016). Furthermore, patients suffering from bipolar disorder treated with lithium have longer telomeres, not only in comparison to non-lithium treated patients with bipolar disorder, but also with non-affected relatives (Squassina et al., 2016; Powell et al., 2018). These results suggest that lithium may have many non-autophagic related and pleiotropic anti-aging mechanisms.

Three non-licensed nutraceutical compounds, trehalose, spermidine, and urolithin A have been shown to increase autophagy. Trehalose is a disaccharide used by some species as a mechanism for storing excess sugar to protect against environmental stressors (Sarkar et al., 2007a; Seo et al., 2018). It activates autophagy via a poorly defined mTOR-independent mechanism, and it also acts as chemical chaperone (Ravikumar et al., 2010). It increases clearance of Huntington’s disease-associated polyglutamine expansion aggregates in an *atg-5*-dependent manner in mammalian cell lines (Sarkar et al., 2007b). When fed to *C. elegans*, or when trehalose production is genetically enhanced by shifting storage away from glycogen, it increases lifespan (Honda et al., 2010; Seo et al., 2018) with an upregulation of several well-defined markers of autophagy induction in worms (Seo et al., 2018). Lifespan extension and upregulation of *atg-9* mRNA levels are DAF-16/FOXO-dependent (Seo et al., 2018). Moreover, the longevity of worms with higher trehalose circulation requires LGG-1/Atg8 and Beclin (Seo et al., 2018), thus demonstrating by genetic epistasis that autophagy is required for trehalose to extend lifespan.

Spermidine is a polyamine that extends the lifespan of yeast, worms, flies and mice (Eisenberg et al., 2009, 2016). In worms, the lifespan-extending effects are dependent on intact Beclin, and in flies on Atg7, demonstrating that autophagy is required for the longevity benefits of spermidine (Eisenberg et al., 2009). In mice, the cardioprotective functions of spermidine were genetically shown to be dependent on intact autophagy. Furthermore, in humans, higher spermidine intake by questionnaire-assessment was associated with lower levels of heart failure (Eisenberg et al., 2016) and a reduction in all-cause mortality in an Italian population (Kiechl et al., 2018).

Urolithin A is one of the three end-products (the other two being Urothilin B and C) of microflora-mediated processing of ellagic acid in the gut from ellagitannins. It is contained in berries, acorns, nuts and tree leaves. When fed to *C. elegans*, urolithin A extends lifespan and improves healthspan (Ryu et al., 2016), and reduces Aβ-associated memory loss (Fang et al., 2019). The lifespan-extending properties of urolithin A are at least partially dependent on AMPK, and completely dependent on intact mitochondria, as it induces mitophagy (Ryu et al., 2016). The worm lifespan extension produced by urolithins was completely dependent on Pink1, *dct-1*/BNIP3, Beclin, *pdr-1*/Parkin, *sqst-1*, *vps-34* and *skn-1*/Nrf1/2. In mice, urolithin A treatment has also been linked to mitophagy induction and improved muscle function (Ryu et al., 2016).

Among the autophagy-activating drugs with anti-aging effects, rapamycin is the best characterized. It is licensed for clinical use in humans as a co-immunosuppressant in renal transplantation. It is also used to coat coronary stents in the prevention of restenosis following coronary angioplasty, and to treat lymphangioleimyomatosis, a rare lung disease of smooth muscle cell growth. Rapamycin is the flagship mTORC1 inhibitor and acts by allosterically binding to FK506-binding protein12 (FKBP12) to form a complex, which in turn binds and inhibits mTOR (Grolleau et al., 2002; Thoreen et al., 2009). Rapamycin has been shown to extend lifespan in yeast, worms, flies and mice (Powers et al., 2006; Medvedik et al., 2007; Harrison et al., 2009; Bjedov et al., 2010; Robida-Stubbs et al., 2012; Miller et al., 2014), and a randomized controlled preclinical trial in dogs is in the pipeline to study its anti-aging effects (Urfer et al., 2017). In addition, rapamycin treatment in mice and elderly people has been linked to an improved response to vaccination against influenza virus (Chen et al., 2009; Mannick et al., 2014, 2018). The mechanism of action of rapamycin-mediated lifespan extension has been shown to be independent of dietary restriction in flies (Bjedov et al., 2010), and metabolically and transcriptionally different in mice (Miller et al., 2014). While autophagy is assumed to be upregulated when mTOR is inhibited (either genetically or pharmacologically), so far and to the best of our knowledge, the only epistatic investigation showing that autophagy is required for lifespan extension has been performed in flies (Bjedov et al., 2010). However, rapamycin-mediated lifespan extension also requires S6K (Bjedov et al., 2010), another downstream effector important in translation and ribosomal physiology (Pende et al., 2004; Chauvin et al., 2014). In addition, in *C. elegans* and *Drosophila*, rapamycin-mediated lifespan extension does not require DAF-16/FOXO (Bjedov et al., 2010; Robida-Stubbs et al., 2012), but instead requires the redox regulator SKN-1/Nrf1/2 in worms (Robida-Stubbs et al., 2012). The effects of rapamycin are mostly specific to mTORC1. However, under certain conditions of prolonged exposure, it can also inhibit mTORC2 (Sarbassov et al., 2006). In rodents and patients, rapamycin leads to insulin resistance and hyperlipidemia (Morrisett et al., 2002; Houde et al., 2010), and this has been attributed to its effects on mTORC2 inhibition (Lamming et al., 2012). Interestingly, in *Drosophila* higher triacylglycerol content associated with rapamycin treatment is abolished in combination with lithium, and pronounced lifespan extension is achieved when a combination of three drugs, rapamycin, lithium, and trametinib is fed to flies (Castillo-Quan et al., 2019). Efforts continue in developing dual mTORC1/mTORC2 catalytic inhibitors like the Torin1 and Torin2 (Thoreen et al., 2009), which have already been shown to reduce cellular senescence in mammalian cells and extend lifespan in *Drosophila* (Leontieva and Blagosklonny, 2016; Mason et al., 2018). However, the reality is that we need to identify new compounds that specifically target the autophagy process. Otherwise, the non-autophagy-related effects of inhibiting mTORC1, mTORC2 or other pathways will not allow a clear examination of the potentially beneficial clinical effects of autophagy activation.

## Conclusion and Future Outlook

In conclusion, we have described the intricate relationship between aging and autophagy, and discussed major anti-aging interventions that depend on autophagy enhancement. There is increasing evidence that boosting autophagy flux and the recycling of damaged cellular components may prove to be an effective anti-aging strategy. However, we need to be mindful of the fact that autophagy is a degradative process, and that strong upregulation may therefore be detrimental to the organism. The more evidence we gather for improvement of health during aging by targeting autophagy, the more complexities we are uncovering. Numerous questions are continually arising concerning the optimal manipulation of autophagy that is required to benefit a given organism. These include factors relating to the intensity of autophagy increase, its timing and effect in the young versus old individuals, whether non-selective autophagy should be targeted or if specific cargoes should be selectively degraded. Finally, the question of tissue specificity needs to be addressed, to determine whether autophagy offers greater benefits when augmented in a specific single tissue or in a combination of tissues. Furthermore, despite tremendous progress in the fields of autophagy and aging, in order for successful therapies to be developed, we need to improve our measurements of autophagy flux. By determining which parts of this multistep process are failing in aging and disease, we can tailor our interventions accordingly. One of the main challenges will be to develop specific strategies that either alter selective autophagy, or restore rate-limiting steps in the autophagy process. Many of the current autophagy activators that have been characterized also affect other intracellular processes, such as the inhibition of translation by rapamycin. It is currently unclear whether autophagy-specific drugs, or those targeting multiple cellular pathways, will provide the most potent health benefits. In order to optimize the benefits of autophagy, short temporary treatment may offer the greatest advantages, cleansing the cell of damaged components, while minimizing toxic side-effects. Similar approaches also apply to senolytic drugs that remove senescent cells from an organism. In summary, autophagy has been implicated in a plethora of essential cellular processes, including those involved in the DNA damage response, immunity, cell death and senescence, and thus has critical importance in the identification of new drugs and strategies to improve healthy aging.

## Author Contributions

ES, JC-Q, VM, CL, RK, KK, and IB wrote the manuscript. IB prepared the figure.

## Conflict of Interest

The authors declare that the research was conducted in the absence of any commercial or financial relationships that could be construed as a potential conflict of interest.
